# The association between pancreatic enzyme serum concentrations and blunt pancreatic trauma: a systematic review

**DOI:** 10.1007/s00068-026-03263-9

**Published:** 2026-07-21

**Authors:** Blake Sykes, Dylan Gracias, Cino Bendinelli, Zsolt J. Balogh

**Affiliations:** 1 Department of Surgery, Bankstown Hospital, Sydney, NSW Australia; 2 Department of Surgery, Royal Darwin Hospital, Darwin, NT Australia; 3 Department of Traumatology, John Hunter Hospital, Newcastle, NSW Australia; 4 Discipline of Surgery, School of Medicine and Public Health, University of Newcastle, Newcastle, NSW Australia; 5 Injury and Trauma Research Program, Hunter Medical Research Institute, Newcastle, NSW Australia

**Keywords:** Pancreatic trauma, Blunt abdominal trauma, Lipase, Amylase

## Abstract

**Purpose:**

Blunt pancreatic trauma is uncommon. When suspected, serum amylase and lipase are measured, but their diagnostic value remains uncertain. This systematic review evaluated the diagnostic performance of serum amylase and serum lipase in detecting blunt pancreatic trauma in adults.

**Methods:**

MEDLINE, Embase, Scopus and Cochrane Central were searched for studies evaluating serum amylase and/or lipase in adults with blunt pancreatic trauma. Observational studies reporting diagnostic accuracy or clinical outcomes were included. Two reviewers independently screened studies and assessed risk of bias using the Newcastle-Ottawa Scale.

**Results:**

Four observational studies met inclusion criteria. A total of 4937 patients with blunt abdominal trauma, including 150 with pancreatic injury were investigated. Two studies evaluated serum lipase alone and two compared amylase and lipase. Pancreatic injuries were graded according to the American or Japanese Association for the Surgery of Trauma. Early serum amylase measurement (< 90 min post-injury) demonstrated low sensitivity. In contrast, testing performed more than 3 h after injury has shown improved diagnostic accuracy with one study suggesting combined serum amylase and lipase testing can achieve a sensitivity of 85% and specificity of 100%.

**Conclusion:**

Early serum pancreatic enzymes measurement does not appear to aid initial management decisions following blunt abdominal trauma. Delayed testing (> 3–6 h), particularly using lipase or combined lipase and amylase showed improved diagnostic performance. Prospective multicentre validation is needed to define their role in guiding further imaging after equivocal or negative initial CT.

## Introduction

Blunt pancreatic trauma is uncommon but burdened by high morbidity and mortality [1–4]. Some studies estimate mortality of blunt pancreatic injury to reach 25% [5–7]. The challenging diagnosis is based on clinical signs, radiologic findings and laboratory tests. Serum lipase and serum amylase are used interchangeably in blunt abdominal trauma without well-defined roles to diagnose or exclude pancreatic injury. As such, it is unclear which one is of greater utility in this setting [8–9].

Pancreatic injury results in the premature activation of zymogens (inactive enzyme precursors), causing release of amylase and lipase, and the subsequent locoregional destruction of surrounding tissues. This pathophysiological process is further exacerbated when there is pancreatic ductal disruption [10–12]. Drawing from the broader literature on pancreatitis, serum lipase is favoured for its higher sensitivity and longer half-life [13], and as such, is often considered a superior test [14]. Despite their usual role as screening tools, pancreatic enzymes are burdened by frequent false positive results that may lead to unnecessary imaging or procedures, and some false negative results that may lead to delayed diagnosis of a potentially fatal injury. This systematic review aims to determine the clinical utility of serum amylase and lipase measurement in the acute assessment of blunt trauma patients for the diagnosis of pancreatic injury.

## Methods

A systematic search of the literature was performed to identify interventional and observational studies on blunt pancreatic trauma in adults. MEDLINE, Embase, Scopus and Cochrane Central were searched without limits for date, but with filters for articles pertaining to humans only. Medical subject headings (MeSH) and key words used in the search terms included: (“trauma”) AND (“pancreatic injury” OR “pancreatic trauma” OR “of the pancreas”) AND (“lipase” OR “amylase”) AND (“diagnosis” OR “CT” OR “surgery”). The results were exported to EndNote as a referencing system and de-duplicated manually and then screened according to Preferred Reporting Items for Systematic reviews and Meta-Analysis guidelines (PRISMA) with Covidence using the following inclusion and exclusion criteria.

Only papers that discussed blunt abdominal trauma and suspected pancreatic injury with reference to serum lipase and/or amylase were included. Excluded studies were those addressing penetrating trauma, and those with pre-existing pancreatic pathology including cancer, pancreatitis and transplant patients. Studies regarding the management of pancreatic injury in a paediatric population were also excluded. Opinion pieces, textbook entries, case studies and case series were also excluded.

**Fig. 1:**
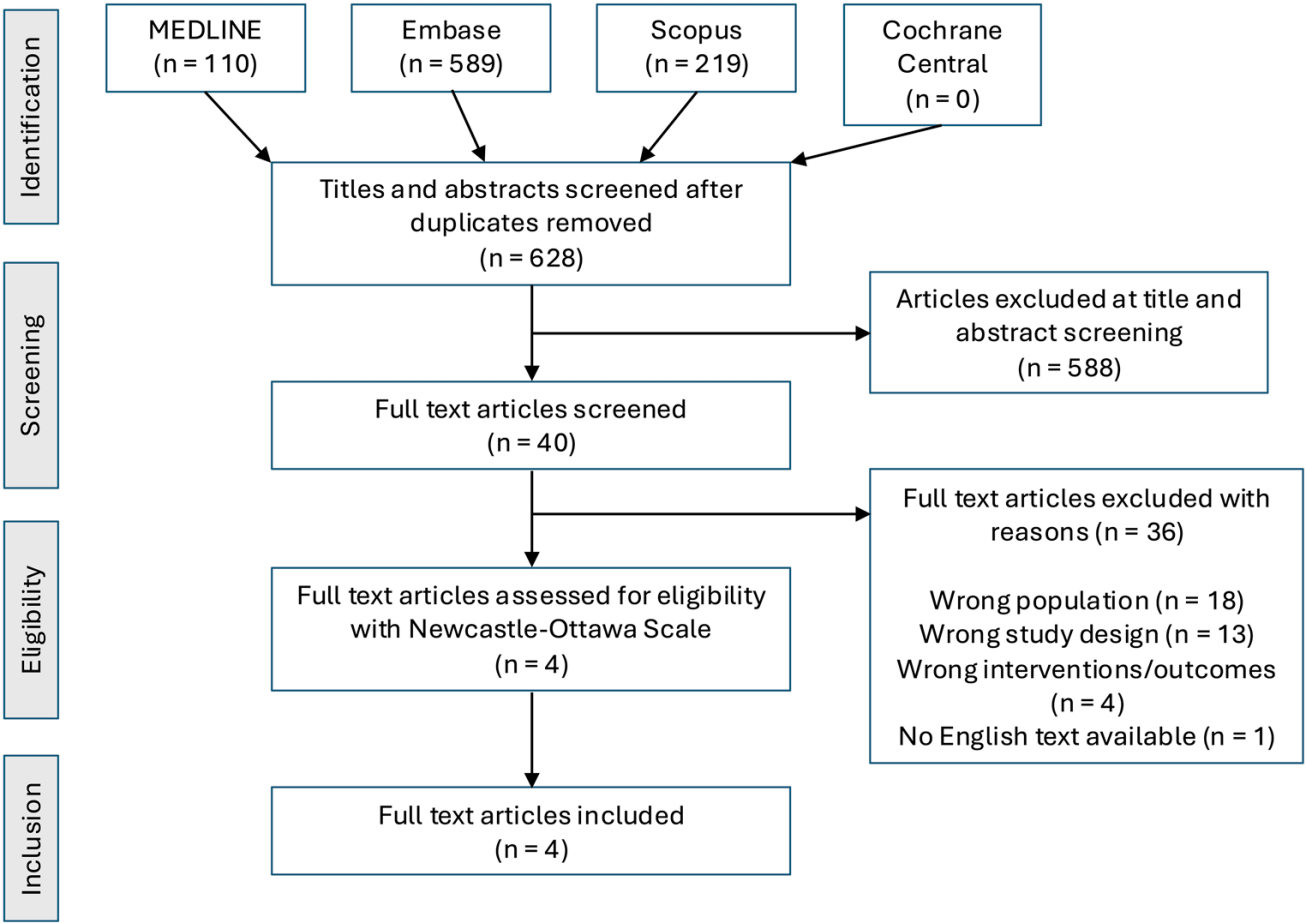
PRISMA flow diagram

Title and abstract screening were performed by two independent authors (BS and DG). A third author then resolved any disagreements (ZJB). Full text review was then completed by the same two independent authors. The reference lists of included publications were then assessed to identify any additional relevant articles, with no other studies being included from this source. See Fig. 1 for the PRISMA flow diagram.

Included articles were then evaluated by the primary author according to the Newcastle-Ottawa Scale for Cohort studies (see Table 1). This assessment tool was chosen as the included articles were all observational studies. Due to their heterogeneity in methodologies and resultant variation in reporting, a meta-analysis was not performed. Data pertaining to baseline patient characteristics were extracted including age, sex, comorbidities, recorded vital signs and mechanism of injury. Data was extracted regarding method of diagnosis of pancreatic injury, grade of injury and subsequent management.

**Table 1:** Newcastle-ottawa quality assessment scale

Articles	Selection				Comparability	Outcome			Score
	Representative of the present cohort	Selection of non-exposed	Ascertainment of exposure	Outcome not present at the start		Assessment of outcome	Adequate follow-up length	Adequate follow-up	
Boulanger, (1993) [15]	★	★	★	★		★	★	★	7 (High)
Takishima, (1997) [16]			★	★		★	★	★	5 (Fair)
Mahajan, (2014) [17]	★	★	★	★	★	★	★	★	8 (High)
Hosseininejad, (2020) [8]		★	★	★		★	★	★	6 (High)

## Results

Four studies met the inclusion criteria for this systematic review: Boulanger et al. (1993) [15], Takishima et al. (1997) [16], Mahajan et al. (2014) [17] and Hosseininejad et al. (2020) [8]. These have been summarised in Table 2. Together, these studies represent a cumulative sample of more than 4600 patients with blunt trauma, though the studies varied widely in design, size and diagnostic approach.

Boulanger et al. retrospectively analysed 4316 unselected blunt trauma admissions in North America, measuring serum amylase within 90 min of presentation, with only 17 patients being diagnosed with pancreatic injury. A Japanese study by Takishima et al. also retrospectively measured serum amylase, however they limited their cohort to 73 patients with confirmed blunt pancreatic injury, examining the time elapsed between injury and amylase result over a 16 year-period. Mahajan et al. compared serum amylase and lipase levels in 164 patients presenting with blunt abdominal trauma, among whom 33 had confirmed pancreatic injuries. In a prospective cohort of 384 blunt abdominal trauma patients, Hosseininejad et al. measured both serum amylase and lipase, identifying pancreatic injury in 27 cases.

There was a variety of enzyme threshold levels used across the four studies, with Boulanger et al. dichotomising serum amylase into normal (< 125 U/L) and abnormal results (≥125 U/L); with 9.2% of their large cohort having an abnormal amylase result. They then sub-grouped these into 126–200 U/L, 201–300 U/L and > 300 U/L. Similarly, due to changes in the assay, Takishima et al. also dichotomise amylase results to normal and raised, with 83.5% having a raised serum amylase result overall. Mahajan et al. also sub-categorised the serum enzyme results for lipase and amylase into categories (≤ 100 IU/L, 100–250 IU/L and ≥ 250 IU/L).

Pancreatic injury was confirmed by Computerised Tomography (CT), Magnetic Resonance Imaging (MRI), Endoscopic Retrograde Cholangiopancreatography (ERCP) or surgical exploration across the four studies. In unselected blunt abdominal trauma cohorts, there was a wide range of incidence of pancreatic injury from < 1% to 21%. Pancreatic injury was graded using the American Association for the Surgery of Trauma (AAST) in two of the studies, with Takishima et al. using a scale from the Japanese Association for the Surgery of Trauma. Hosseininejad et al. did not grade the pancreatic injuries. Amylase correlated poorly with AAST grading in the study by Boulanger et al., with both patients with grade III and IV injuries showing normal serum amylase levels at 1.5 h post injury. Takishima et al. conversely noted a positive correlation between amylase results and severity of injury.

Timing of blood collection after trauma correlated with raised serum enzymes. Boulanger et al. report hyperamylasaemia within 90 min of admission to have a low sensitivity of 35.3% and a positive predictive value (PPV) of 1.5%. Takishima et al. report that when serum amylase is measured within 3 h of injury, 24% of patients with pancreatic injury still had normal results, however after 3 h of injury, all patients (*n* = 24) had raised serum amylase. When the test was delayed, they noted a correlation between injury severity and serum amylase (Type 1 (contusion) injury 259.3 U/L ± 50.8 versus Type 3 (ductal) injury 675 U/L ± 185.2 *p* < 0.05). Mahajan similarly report that all patients with pancreatic injury had positive lipase and amylase after 3 h had elapsed from injury. Equally, all patients with pancreatic injury but with normal serum enzyme results, had their blood test within 2 h of injury. This leads them to report a statistically significant association between serum amylase and lipase elevation and time elapsed from injury to admission (*p* < 0.0001).

Boulanger et al. detected a specificity of 91.3% for hyperamylasaemia in their cohort of 17 patients with blunt pancreatic injury. After logarithmic transformation, Mahajan et al. found that although raised serum amylase levels are often seen in the context of bowel injury, they only become statistically significantly when combined with pancreatic injury (*p* < 0.001). With serum lipase, they identified higher specificity for pancreatic injury (mean 944 IU/L (CI 95% 365–1211), with largely normal lipase results in bowel injury alone (mean of 42 IU/L (CI 95% 30–45). They summarised that serum amylase was highly sensitive (85%, CI 67–94%) and moderately specific (79%, CI 70–85%) with a 50% PPV and a 95% negative predictive value (NPV) for pancreatic injury. Comparatively, their study finds that lipase was both sensitive (85% CI 67–94%) and specific (99% CI 95–99%) with a 97% PPV and 96% NPV for pancreatic injury. However, when serum amylase and lipase are combined 3 h post-trauma, sensitivity remained 85%, but specificity reached 100% (CI 96–100%) with 100% PPV and 96% NPV.

**Table 2:** Summary of included studies

Article	Study design	Serum enzyme used	Pancreatic injury incidence	Summary of findings
Boulanger, (1993) [15]	Retrospective cohort study of blunt abdominal trauma patients	Amylase	17 patients with pancreatic injury, total 4316 patients	Acute hyperamylasaemia (< 90 min after admission) is a poor marker of pancreatic injury in blunt abdominal trauma.
Takishima, (1997) [16]	Retrospective analysis of patients with confirmed pancreatic injury	Amylase	73 patients all with pancreatic injury	The major factor that influences the serum amylase result in blunt pancreatic trauma is time, with serum amylase being non-diagnostic within 3 h of trauma.
Mahajan, (2014) [17]	Prospective cohort study of blunt abdominal trauma patients	Amylase and lipase	33 patients with pancreatic injury, total 164 patients	Combined serum amylase and lipase showed 100% specificity and 85% sensitivity in predicting pancreatic injury. Raised serum lipase and amylase showed significant association with time elapsed since injury.
Hosseininejad, (2020) [8]	Prospective diagnostic study of blunt abdominal trauma patients	Amylase and lipase	27 patients with pancreatic injury, total 384 patients	Pancreatic injury in blunt trauma is associated with a significant increase in levels of serum amylase and lipase.

## Discussion

The utility of serum amylase in trauma has been debated since 1943, with Naffzinger et al. being one of the first to describe a case series of elevated serum amylase in blunt trauma patients [18]. Barnett et al. then later reported that serum amylase levels were not a dependable sign of specific pancreatic trauma [19]. Serum lipase was not investigated as a marker of pancreatic trauma until the 1990s [20]. Confirmation of pancreatic injury also used to rely on positive diagnostic peritoneal lavage (DPL), which owing to modern imaging techniques is no longer widely used. Substantial variation in study populations, together with progressive changes in diagnostic techniques, has contributed to the conflicting results reported in the literature over several decades.

The inconsistent findings across the existing literature create uncertainty about the reliability of these markers in the trauma setting. The only existing *multicentre* study comparing the utility of these enzymes is in a paediatric population with conclusions that are not particularly favourable of their use as they report that high-grade pancreatic injuries were not predicted by the early use of amylase and lipase in trauma [21]. Studies also report that in a paediatric population; these enzymes are not cost-effective as stand-alone screening tests for blunt pancreatic trauma, nor do they correlate with grade of injury [21–22].

The utility of pancreatic enzymes appears to depend on the timing of the blood test. The enzymes levels in patients with subsequently confirmed pancreatic injury may be normal for several hours after blunt trauma. Across the study designs, delayed blood tests improve the sensitivity for both enzymes. This is similar to what is described in the paediatric literature, where Matsuno et al. suggest an improved positive correlation between serum amylase and blunt pancreatic trauma in a cohort of 51 children [23]. These studies suggest that a single admission blood test of either enzyme, especially less than three hours post-injury, has limited predictive value and as such should be avoided. Where pancreatic enzymes remain persistently negative, the strong negative predictive value reported by Mahajan et al. would refute pancreatic injury and would suggest that no further imaging is required.

From this review, biological plausibility of the enzymes’ behaviour appears to support the empirical observations that amylase is produced not only by the pancreas, but also by the salivary gland and can be raised in a wide range of conditions including head injury, shock, renal impairment and bowel trauma [24–27]. This may explain why hyperamylasaemia often reflected injuries remote from the pancreas in the study from Boulanger et al. Lipase however, is more pancreas-specific and with a longer half-life of 6.7 to 13.7 h, which may suggest that positive results persist longer, aiding in detection in the delayed presenter [13]. This may explain the findings of Mahajan et al., where delayed lipase elevation almost always corresponded to pancreatic injury.

This systematic review highlights the lack of standardised diagnosis and grading of pancreatic injury, with a combination of modalities used across the studies, including CT, MRI, ERCP and surgical exploration, and two different grading systems used. As such, this variability in the reference standards affects the comparability and validity of any diagnostic estimates that can be drawn. The study is also limited by the small number of studies concerning the study topic, the heterogeneity in study design and the fact that some were 25 years old, with significant change in clinical practice across this timeframe. There is also a relative paucity in the data for adults, compared to studies in paediatric populations. Recognising the differences in mechanisms of trauma, physiology and body composition, this results in difficult extrapolation of data from studies pertaining to paediatric trauma, leading to their exclusion in this systematic review.

Regardless of the limitations, this systematic review has shed light on the pragmatic use of these laboratory tests. The selective use of pancreatic enzymes in the context of negative initial imaging with strong clinical concern for pancreatic injury after 3–6 h may help to guide repeat imaging to aid diagnosis. Due to the scarcity of relevant studies, this concept would need to be validated with a prospective multicentre study comparing both enzymes in blunt abdominal trauma in an adult population.

## Conclusion

This systematic review suggests that early post-injury serum pancreatic enzyme measurement is unlikely to add to clinical decision making on imaging or management of patients with blunt abdominal trauma. After 3–6 h lipase or combined lipase and amylase tests are always positive in pancreatic injuries with 85% sensitivity. Similarly, persistently normal pancreatic enzyme results would be of utility in refuting the diagnosis of pancreatic injury and can help to rationalise the use of serial imaging where initial imaging has not been diagnostic. This potential later diagnostic utility of pancreatic enzymes requires prospective multicentre validation to guide the potential reimaging or further imaging of patients with initially negative or suspicious CT scans for pancreatic injury.

## Data Availability

No datasets were generated or analysed during the current study.
